# Plasma fatty acid levels and gene expression related to lipid metabolism in peripheral blood mononuclear cells: a cross-sectional study in healthy subjects

**DOI:** 10.1186/s12263-018-0600-z

**Published:** 2018-04-10

**Authors:** Sunniva V. Larsen, Kirsten B. Holven, Inger Ottestad, Kine N. Dagsland, Mari C. W. Myhrstad, Stine M. Ulven

**Affiliations:** 1 Department of Nutrition, Institute for Basic Medical Sciences, University of Oslo, P.O. Box 1046, Blindern, 0317 Oslo, Norway; 2 Norwegian National Advisory Unit on Familial Hypercholesterolemia, Department of Endocrinology, Morbid Obesity and Preventive Medicine, Oslo University Hospital, P.O. Box 4950, Nydalen, 0424 Oslo, Norway; 3 Department of Health, Nutrition and Management, Faculty of Health Sciences, Oslo and Akershus University College of Applied Sciences, P.O. Box 4, St. Olavs plass, 0130 Oslo, Norway

**Keywords:** Cardiovascular risk factors, Nutrition, Fat quality, Plasma fatty acids, Lipid metabolism, Gene expression, Peripheral blood mononuclear cells

## Abstract

**Background:**

Solid evidence indicates that intake of marine n-3 fatty acids lowers serum triglycerides and that replacing saturated fatty acids (SFA) with polyunsaturated fatty acids (PUFA) reduces plasma total cholesterol and LDL cholesterol. The molecular mechanisms underlying these health beneficial effects are however not completely elucidated. The aim of this study was therefore to investigate the expression of genes related to lipid metabolism in peripheral blood mononuclear cells (PBMC) depending on the plasma levels of n-6 and n-3 fatty acids and the SFA to PUFA ratio.

**Methods:**

Fifty-four healthy subjects were grouped into tertiles (*n* = 18) based on plasma levels of n-6 and n-3 fatty acids and the SFA to PUFA ratio. The PBMC gene expression levels among subjects in the highest versus the lowest tertiles were compared. In total, 285 genes related to cholesterol and triglyceride metabolism were selected for this explorative study.

**Results:**

Among the 285 selected genes, 161 were defined as expressed in the PBMCs. The plasma SFA to PUFA ratio was associated with the highest number of significantly different expressed genes (25 gene transcripts), followed by plasma n-6 fatty acid level (15 gene transcripts) and plasma n-3 fatty acid level (8 gene transcripts). In particular, genes involved in cholesterol homeostasis were significantly different expressed among subjects with high compared to low plasma SFA to PUFA ratio.

**Conclusion:**

Genes involved in lipid metabolism were differentially expressed in PBMCs depending on the plasma fatty acid levels. This finding may increase our understanding of how fatty acids influence lipid metabolism at a molecular level in humans.

**Electronic supplementary material:**

The online version of this article (10.1186/s12263-018-0600-z) contains supplementary material, which is available to authorized users.

## Background

Cardiovascular disease (CVD) is the leading cause of morbidity and mortality worldwide [1]. Dyslipidemia, including elevated levels of plasma total cholesterol (total-C), low-density lipoprotein cholesterol (LDL-C), and triglycerides (TG), is a major risk factor for CVD. Dietary fatty acids play a significant role in modulating plasma lipids, thereby influencing the CVD risk [2]. Solid evidence indicates that intake of marine n-3 fatty acids, and replacing saturated fatty acids (SFA) with polyunsaturated fatty acids (PUFA), prevents CVD and CVD mortality [3–7]. One of the CVD reducing effects of marine n-3 fatty acids is the TG lowering effect, while replacing SFAs with PUFAs has been shown in several randomized controlled trials to reduce plasma total- and LDL-C [8–10]. Animal studies and in vitro experiments have demonstrated different molecular mechanisms of how marine n-3 fatty acids reduce hepatic very low-density lipoprotein (VLDL) production and increase the VLDL clearance [11, 12]. The molecular mechanisms behind the total- and LDL-C lowering effects of replacing SFAs with PUFAs are however less clear. Therefore, studies investigating the molecular mechanisms underlying the health effects of SFAs and PUFAs in humans are warranted.

In humans, linoleic acid (LA; 18:2n-6) and alpha-linolenic acid (ALA; 18:3n-3) are not biosynthesized de novo. Since the conversion of these fatty acids into long chain PUFAs is limited, plasma PUFA levels have been shown to be objective biomarkers of dietary intake [13, 14]. Hence, using plasma fatty acids is an alternative approach to examine the association between dietary fat quality and CVD risk.

The ability of fatty acids to regulate gene transcription may account for their effects on lipid metabolism. Fatty acids regulate gene transcription directly by binding as ligands to specific transcription factors, thereby controlling the activity of the transcription factor, or indirectly by regulating different signaling pathways controlling the nuclear abundance of transcription factors [15–17]. In particular, there is considerable evidence that PUFAs modulate the transcription of genes involved in lipid metabolism by regulating the activity of the nuclear receptors peroxisome proliferator activated receptors (PPAR) and liver x receptors (LXR) or by suppressing the nuclear abundance of the sterol regulatory binding proteins (SREBP) [17, 18]. Our understanding of how SFAs modulate the expression of genes encoding proteins related to lipid metabolism is however more limited [19].

In order to get a comprehensive understanding of how dietary fat quality affect lipid metabolism, to prevent dyslipidemia in humans, we need a suitable model system. Changes in gene expression occur prior to changes in protein levels, and gene expression analysis is therefore a valuable and sensitive technique measuring early changes related to diet [20, 21]. Peripheral blood mononuclear cells (PBMC) include lymphocytes and monocytes which circulate around in the body and are exposed to both environmental factors and metabolic tissues. Studies have shown that PBMCs may be used as a surrogate model for liver metabolism since these cells reflect hepatic regulation of cholesterol metabolism as well as metabolic and immune responses [22–26].

Some postprandial studies have examined the effect of fat intake on the mRNA level of genes involved in lipid metabolism in PBMCs [26, 27]. To our knowledge, no studies have particularly focused on the impact of plasma fatty acid levels on PBMC gene expression related to lipid metabolism. The aim of the present study was therefore to investigate the relation of plasma levels of n-6 and n-3 fatty acids, and SFA to PUFA ratio, to PBMC gene expression specifically related to lipid metabolism using cross-sectional data from a human intervention study [28].

## Methods

### Study design and participants

Fifty-four healthy, non-smoking men and women aged 18–50 years were included in this cross-sectional sub-study of a randomized controlled trial designed to investigate the health effects of fish oil with different quality focusing on lipids, oxidative stress, and inflammation [28, 29]. In addition, we have analyzed the plasma lipidomic profile, the PBMC gene expression profile, and the effects on lipoprotein subclasses from this dietary intervention study [30–33]. A detailed description of the protocol, participant recruitment and enrolment, inclusion and exclusion criteria, and compliance is described elsewhere [28]. In the present study, data from the end of intervention was utilized.

The study population was grouped into tertiles three times according to the plasma fatty acid levels and the SFA to PUFA ratio by arranging samples from the highest to the lowest value. First, the subjects were grouped according to the plasma level of total n-3 fatty acids which included ALA, eicosapentaenoic acid (EPA; 20:5n-3), docosapentaenoic acid (DPA; 22:5n-3), and docosahexaenoic acid (DHA; 22:6n-3). Second, the subjects were grouped according to the plasma level of total n-6 fatty acids which included LA and arachidonic acid (AA; 20:4n-6). Finally, the subjects were grouped according to the plasma SFA to PUFA ratio, which included the SFAs myristic acid (14:0), palmitic acid (16:0), and stearic acid (18:0), and the PUFA included the sum of plasma total levels of n-3 and n-6 fatty acids. The subjects in the highest (*n* = 18) and the lowest (*n* = 18) tertile were compared.

The intervention study was conducted according to the guidelines laid down in the Declaration of Helsinki, and all procedures involving human subjects were approved by the Regional Committee of Medical Ethics (approval no. 6.2008.2215) and the Norwegian Social Science Data Services (approval no. 21924). Written informed consent was obtained from all participants. The study was registered at www.clinicaltrials.gov (ID no. NCT01034423).

### Clinical and biochemical measurements

Procedures regarding clinical and biochemical measurements have been described earlier [28]. In brief, fasting venous blood samples were collected after an overnight fast (≥ 12 h). Serum was obtained from silica gel tubes (Becton Dickinson Vacutainer Systems, UK) and kept at room temperature for 30 min before centrifugation (1500*g*, 12 min). Plasma was obtained from EDTA tubes (Becton Dickinson Vacutainer Systems, UK), immediately placed on ice and centrifuged within 10 min (1500*g*, 4 °C, 10 min). EDTA tubes with whole blood were kept at room temperature for a maximum of 48 h before counting the total number of lymphocytes and monocytes. Fasting serum concentrations of total-C, LDL-C, HDL-C, and TGs, as well as lymphocyte and monocyte counts, were measured by standard methods in a routine clinical laboratory (Fürst Medical Laboratory, Oslo, Norway).

### Fatty acid analysis

Plasma lipids were extracted by use of the Bligh and Dyer method [34], as described by Ottestad et al. [28]. Fatty acids in Bligh and Dyer extract were derivatized and analyzed as methyl esters on a GC (HP 6890) equipped with a BPX-70 column (SGE Analytical Science Private Limited, Melbourne, Australia). The plasma level of the individual fatty acids is expressed as mass percentage (%wt) of total fatty acids in plasma.

### PBMC and RNA isolation

PBMCs were isolated by using the BD Vacutainer Cell Preparation tubes according to the manufacturer’s instructions (Becton, Dickinson San Jose, CA, USA), as described previously [31]. This is a well-documented and standardized method to collect mononuclear cells with high purity (above 90%), and according to the manufacturer, approximately 80% of the cells are lymphocytes and 12% are monocytes. Pellets were stored at − 80 °C for further RNA analysis. Total RNA was isolated from the PBMC samples using RNeasy Mini Kit (Qiagen). RNA quantity and quality were measured using the ND 1000 Spectrophotometer (Seven Werner AB) and Agilent bioanalyser (Agilent Technologies Inc.), respectively.

### Microarray analysis and selection of genes

Gene expression was analyzed by hybridization to an Illumina HumanHT-12 v4 Expression BeadChip and scanned on an Illumina HiSCan microarray scanner (Illumina Inc., CA 92122). Illumina GenomeStudio was used to transform bead-level data to probe-level intensities and statistics, which were exported raw for bioinformatics analysis. Quantile normalization of the Illumina intensities was performed, and probes without a detectable expression (detection *P* > 0.01) in at least 10% of the samples were excluded from further analyses. From the 48,000 probes presented on the Illumina array, 21,236 probes were defined as expressed in the current study. A more detailed description of the protocol is given elsewhere [31]. The raw data are available from the Gene Expression Ominbus (GEO) (accession number GSE111567).

A total of 285 genes encoding proteins related to cholesterol and TG metabolism were selected for this explorative study. The genes were selected based on relevant gene sets related to cholesterol and TG metabolism (26 gene sets) in the Molecular Signature Database v6.0 [35] limited to collection C5 (Gene ontology, GO), as well as literature summarizing loci associated with different lipid traits [36]. Out of the 285 genes, 161 genes were defined as expressed on the HumanHT-12 v4 microarray and included in the statistical analyses (Additional file 1). The list of the differentially expressed genes was based on the lowest *P* values for genes containing multiple probe set. The expression levels of the differentially expressed genes which were expressed by more than one probe are shown in Additional files 2, 3, and 4.

### Statistical analysis

Differences in Log2 gene expression between subjects in the highest and lowest tertiles were compared by independent samples *t* test. No adjustment for multiple testing was performed because of the explorative design of the study. Significantly different expressed genes were further correlated with clinical and biochemical parameters by Pearson’s correlation. Differences in subject characteristics and plasma fatty acid levels between subjects in the highest and lowest tertiles were compared by independent samples *t* test or Mann-Whitney *U* test when normally and not normally distributed, respectively. All statistical analyses were performed using R open source software version 3.4.1 [37]. *P* values < 0.05 were considered significant*.*

## Results

### Subject characteristics and plasma fatty acid levels

The subjects included in the study were young and middle-aged adults with a median age of 25 (22-30) years and BMI (22.6 ± 2.6 kg/m^2^) and serum lipids within the normal range as shown in Table 1. There was a skewed distribution of men and women (15 men and 39 women) among the subjects. The plasma levels of fatty acids in the study population are shown in Table 1.

**Table 1:** Characteristics and plasma fatty acid profile of the study population

	n 54
Male/female (*n*)	15/39
Age (years)	25 (22-30)
BMI (kg/m^2^)	22.7 ± 2.6
Total-C (mmol/l)	4.8 ± 0.9
LDL-C (mmol/l)	2.7 ± 0.8
HDL-C (mmol/l)	1.5 ± 0.4
TG (mmol/l)	0.9 (0.7–1.1)
Plasma level of fatty acids (wt%)	
Total SFA	30.7 (29.5–31.9)
Myristic acid (14:0)	0.9 (0.7–1.1)
Palmitic acid (16:0)	21.5 (20.6–23.3)
Stearic acid (18:0)	7.9 ± 1.0
Total n-6	34.7 ± 3.9
LA (18:2n-6)	28.8 ± 3.6
AA (20:4n-6)	5.9 ± 1.1
Total n-3	6.3 ± 2.4
ALA (18:3n-3)	0.5 (0.5–0.6)
EPA (20:5n-3)	1.94 (0.7–2.7)
DPA (22:5n-3)	0.6 ± 0.2
DHA (22:6n-3)	3.2 ± (2.1–4.1)
Total PUFA	40.1 (38.9–48.1)
SFA to PUFA ratio	0.8 ± (0.7–0.8)

Values are presented as frequency, mean ± SD or median and 25th–75th percentiles

*BMI* body mass index, *Total-C* total cholesterol, *LDL-C* low-density lipoprotein cholesterol, *HDL-C* high-density lipoprotein cholesterol, *TG* triglyceride, *SFA* saturated fatty acid, *LA* linoleic acid, *AA* arachidonic acid, *ALA* alpha-linolenic acid, *EPA* eicosapentaenoic acid, *DPA* docosapentaenoic acid, *DHA* docosahexaenoic acid, *PUFA* polyunsaturated fatty acid

The characteristics and plasma fatty acid levels of subjects in the highest (*n* = 18) and lowest (*n* = 18) tertiles based on plasma levels of n-6 and n-3 fatty acids, and SFA to PUFA ratio, are presented in Table 2. The serum level of TG was significantly lower among subjects in the highest compared to subjects in the lowest plasma n-6 fatty acid tertile (*P* < 0.01). In contrast, the serum level of TG was significantly higher among subjects in the highest compared to subjects in the lowest plasma SFA to PUFA ratio tertile (*P* < 0.01). There were no significant differences in any markers between subjects in the highest and the lowest plasma n-3 fatty acid tertile.

**Table 2:** Characteristics and plasma fatty acid profile of subjects in the highest and lowest tertiles

	n-6 level			n-3 level			SFA/PUFA ratio		
	Highest tertile (n 18)	Lowest tertile (n 18)	*P*	Highest tertile (n 18)	Lowest tertile (n 18)	*P*	Highest tertile (n 18)	Lowest tertile (n 18)	*P*
Male/female	5/13	5/13		3/15	5/13		5/13	6/12	
Age (years)	28 (23–33)	26 (21–29)	0.31	27 (21–31)	27 (21–28)	0.80	27 (22–29)	29 (23–33)	0.38
BMI (kg/m^2^)	22.5 ± 2.9	22.6 ± 2.6	0.94	22.2 ± 2	22.7 ± 3.3	0.57	23.3 ± 2.7	22.6 ± 2.6	0.41
Total-C (mmol/l)	4.9 ± 0.9	4.7 ± 1.1	0.65	4.9 ± 1	4.9 ± 1	0.96	4.7 ± 0.8	5.0 ± 0.9	0.25
LDL-C (mmol/l)	2.7 ± 0.8	2.6 ± 0.9	0.79	2.6 ± 0.9	2.8 ± 0.6	0.71	2.5 ± 0.6	2.8 ± 0.9	0.16
HDL-C (mmol/l)	1.6 ± 0.4	1.4 ± 0.3	0.15	1.6 ± 0.3	1.4 ± 0.4	0.28	1.5 ± 0.4	1.6 ± 0.3	0.74
TG (mmol/l)	0.8 (0.6–0.9)	1.4 (0.9–1.7)	*0.01*	0.9 (0.6–1.1)	1.2 (0.7–1.2)	0.30	1.3 (0.8–1.6)	0.8 (0.6–0.9)	*0.01*
Lymphocytes (%)	38.4 ± 6.8	43.0 ± 8.5	0.08	42.2 ± 10.0	37.6 ± 4	0.09	42.1 ± 8.7	37.4 ± 8.4	0.11
Monocytes (%)	8.5 ± 1.8	8.1 ± 1.9	0.49	8.1 ± 1.7	8.6 ± 2.3	0.42	8.8 ± 2.2	8.48 ± 2.0	0.71
Plasma level of fatty acids (wt%)									
Total SFA	29.6 (28.8–30.5)	32.7 (31.1–33.2)	*< 0.01*	30.7 (29.3–32.3)	31.3 (29.8–31.8)	0.78	33.0 (31.5–33.2)	29.1 (28.7–29.5)	*< 0.01*
Myristic acid (14:0)	0.8 (0.6–1.0)	1.2 (0.8–1.2)	*< 0.01*	0.9 (0.6–1.1)	1.0 (0.7–1.1)	0.57	1.2 (0.9–1.2)	0.7 (0.6–0.9)	*< 0.01*
Palmitic acid (16:0)	20.8 (20.2–21.6)	23.5 (21.4–24.2)	*< 0.01*	21.9 (20.4–23.7)	22.2 (20.9–22.6)	0.39	23.8 (21.8–24.5)	20.7 (20.2–20.7)	*< 0.01*
Stearic acid (18:0)	8.1 ± 0.7	8.1 ± 1.1	0.86	8.2 ± 1.1	8.0 ± 1.0	0.65	8.0 ± 1.1	7.7 ± 0.8	0.37
Total n-6	38.6 ± 1.3	30.3 ± 2.9	*< 0.01*	31.0 ± 4.0	31.5 ± 4.5	0.74	30.8 ± 3.4	38.0 ± 2.0	*< 0.01*
LA (18:2n-6)	32.3 ± 1.6	24.9 ± 2.5	*< 0.01*	27.8 ± 3.8	29.2 ± 4.1	0.29	24.8 ± 2.5	31.8 ± 2.1	*< 0.01*
AA (20:4n-6)	6.2 ± 1.1	5.4 ± 0.9	*0.02*	5.8 ± 0.7	6.2 ± 1.2	0.32	5.9 ± 1.3	6.2 ± 1. 1	0.58
Total n-3	5.8 ± 2.5	7.0 ± 2.3	0.12	9.0 ± 0.9	3.6 ± 0.49	*< 0.01*	6.1 ± 2.5	6.8 ± 2.4	0.44
ALA (18:3n- 3)	0.6 (0.5–0.6)	0.5 (0.4–0.6)	0.46	0.5 (0.5–0.6)	0.5 (0.5–0.6)	0.79	0.5 (0.5–0.6)	0.5 (0.5–0.5)	0.95
EPA (20:5n-3)	1.7 (0.6–2.3)	2.3 (1.8–2.9)	0.14	3.2 (2.8–3.7)	0.6 (0.5–0.6)	*< 0.01*	1.8 (0.7–2.6)	2.2 (1.3–3.2)	0.46
DPA (22:5n-3)	0.6 ± 0.1	0.7 ± 0.2	0.26	0.8 ± 0.1	0.5 ± 0.1	*< 0.01*	0.6 ± 0.2	0.6 ± 0.1	0.80
DHA (22:6n-3)	3.0 (1.9–4.1)	3.6 (3.1–4.1)	0.14	4.3 (4.1–4.7)	1.9 (1.8–2.1)	*< 0.01*	3.2 (2.0–4.1)	3.4 (2.8–4.3)	0.48
Total PUFA	44.4 (42.1–47.3)	37.3 (35.8–39.7)	*< 0.01*	42.6 (39.5–46.7)	40.4 (37.7–41.5)	*0.05*	36.9 (35.8–39.1)	44.7 (43.1–47.3)	*< 0.01*
SFA to PUFA ratio	0.7 (0.6–0.7)	0.8 (0.8–0.9)	*< 0.01*	0.7 (0.6–0.8)	0.8 (0.7–0.8)	0.13	0.9 (0.8–0.9)	0.7 (0.6–0.7)	*< 0.01*

Lymphocytes and monocytes are given as percentage of total white cell count. Differences between tertiles were analyzed using the independent samples *t* test when normally distributed or the Mann-Whitney *U* test when not normally distributed. *P* values < 0.05 were considered significant and are presented in italic. Values are presented as mean ± SD or medians and 25th–75th percentile

*BMI* body mass index, *Total-C* total cholesterol, *LDL-C* low-density lipoprotein cholesterol, *HDL-C* high-density lipoprotein cholesterol, *TG* triglycerides, *SFA* saturated fatty acid, *LA* linoleic acid, *AA* arachidonic acid, *ALA* alpha-linolenic acid, *EPA* eicosapentaenoic acid, *DPA* docosapentaenoic acid, *DHA* docosahexaenoic acid, *PUFA* polyunsaturated fatty acid

As expected, the plasma levels of EPA, DPA, and DHA were significantly higher among subjects in the highest compared to subjects in the lowest plasma n-3 fatty acid tertile (*P* < 0.01, *P* < 0.01, and *P* < 0.01, respectively) (Table 2). The plasma levels of myristic and palmitic acid were significantly higher among subjects in the highest compared to subjects in the lowest plasma SFA to PUFA ratio tertile (*P* < 0.01 and *P* < 0.01, respectively) and significantly lower among subjects in the highest compared to subjects in the lowest plasma n-6 fatty acid tertile (*P* < 0.01 and *P* < 0.01, respectively). In addition, the plasma levels of LA and AA were significantly higher among subjects in the highest compared to subjects in the lowest plasma n-6 fatty acid tertile (*P* < 0.01 and *P* = 0.02, respectively). The percentage of monocytes and lymphocytes did not significantly differ among subjects in the highest compared to the lowest plasma n-3 fatty acid tertile, n-6 fatty acid tertile, and the SFA to PUFA ratio tertile, respectively (Table 2).

### PBMC gene expression

**Fig. 1:**
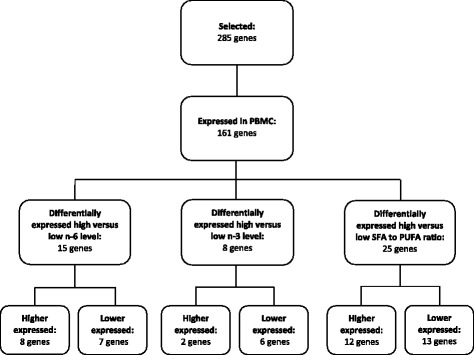
Flowchart of gene selection and number of differentially expressed genes between subjects in the highest and lowest tertile of plasma n-6 level, plasma n-3 level and plasma SFA to PUFA ratio. Differences in Log2 gene expression between subjects in the highest and lowest tertiles were compared by independent samples *t* test. *P* values < 0.05 were considered significant

**Fig. 2:**
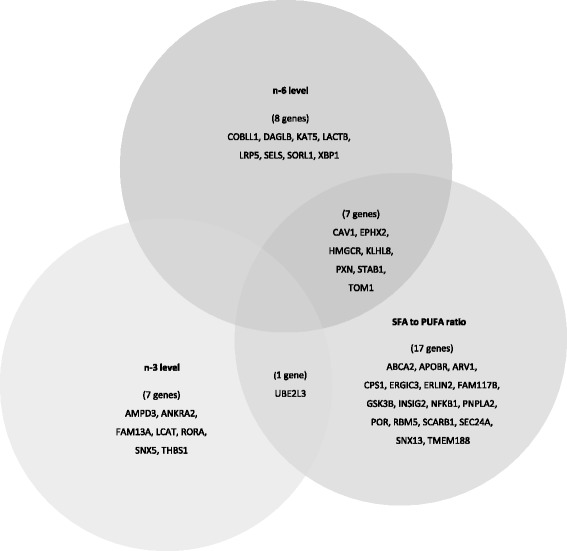
Differently expressed genes associated with plasma fatty acid levels and SFA to PUA ratio. Number of differentially expressed genes associated with n-3 level (8 gene transcripts), n-6 level (15 gene transcripts), and SFA to PUFA ratio (25 gene transcripts). Seven differentially expressed genes were associated with both n-6 level and SFA to PUFA ratio, and one differently expressed gene was associated with both n-3 level and SFA to PUFA ratio. Differences in Log2 gene expression between subjects in the highest and lowest tertiles were compared by independent samples *t* test. *P* values < 0.05 were considered significant

Out of the 161 mRNA transcripts included in the study, 41 were significantly differently expressed depending on plasma fatty acid levels and the SFA to PUFA ratio (Fig. 1). The plasma SFA to PUFA ratio was associated with the highest number of significantly different expressed genes (25 gene transcripts, *P* < 0.05), followed by plasma n-6 fatty acid level (15 gene transcripts, *P* < 0.05), and n-3 fatty acid level (8 gene transcripts, *P* < 0.05), as shown in Fig. 1. Seven gene transcripts were associated with both plasma n-6 fatty acid level and plasma SFA to PUFA ratio, and one gene transcript was associated with both plasma n-3 fatty acid level and plasma SFA to PUFA ratio (Fig. 2). No gene transcripts were shared across plasma n-6 and n-3 fatty acid levels and plasma SFA to PUFA ratio. Differentially expressed genes associated with the plasma levels of n-6 and n-3 fatty acids and the SFA to PUFA ratio are presented in Tables 3, 4, and 5, respectively.

**Table 3:** Differentially expressed genes between subjects in the highest and lowest plasma n-6 tertile

Gene	Highest tertile (n 18)	Lowest tertile (n 18)	Mean difference	*P*
STAB1*	8.66 ± 0.30	8.30 ± 0.47	0.36	0.011
*SORL1*	*10.25 ± 0.27*	*10.06 ± 0.27*	*0.19*	*0.039*
KLHL8*	*9.13 ± 0.25*	*8.94 ± 0.19*	*0.18*	*0.020*
DAGLB	9.21 ± 0.30	9.03 ± 0.19	0.18	0.040
TOM1*	*9.78 ± 0.24*	*9.62 ± 0.20*	*0.16*	*0.037*
PXN*	8.14 ± 0.19	8.00 ± 0.18	0.14	0.030
HMGCR*	8.85 ± 0.14	8.76 ± 0.11	0.09	0.036
*KAT5*	*7.45 ± 0.09*	*7.39 ± 0.09*	*0.06*	*0.047*
LRP5	6.79 ± 0.09	6.87 ± 0.09	− 0.08	0.013
*LACTB*	*7.15 ± 0.12*	*7.24 ± 0.12*	*− 0.09*	*0.027*
*SELS*	*9.66 ± 0.12*	*9.83 ± 0.27*	*− 0.18*	*0.020*
*XBP1*	*10.63 ± 0.23*	*10.85 ± 0.37*	*− 0.22*	*0.042*
EPHX2*	8.18 ± 0.32	8.40 ± 0.30	− 0.22	0.043
CAV1*	6.88 ± 0.11	7.13 ± 0.32	− 0.25	0.005
COBLL1	7.98 ± 0.23	8.28 ± 0.49	− 0.30	0.028

Expression of genes is given as mRNA level. Values are presented as mean ± SD and are log2 transformed. Differences between tertiles were analyzed using the independent samples *t* test. *P* values < 0.05 were considered significant

*Genes differentially expressed between subjects in the highest and lowest n-6 tertile and between subjects in the highest and lowest SFA to PUFA ratio tertile. The genes presented in italics are expressed by more than one probe (shown in Additional file 2: Table S2). The probe with lowest *p*-value is shown in Table 3

**Table 4:** Differentially expressed genes between subjects in the highest and lowest plasma n-3 tertile

Gene	Highest tertile (n 18)	Lowest tertile (n 18)	Mean Difference	*P*
*SNX5*	*8.53 ± 0.13*	*8.43 ± 0.14*	*0.10*	*0.029*
AMPD3	6.94 ± 0.06	6.88 ± 0.06	0.06	0.012
LCAT	6.93 ± 0.07	6.99 ± 0.07	− 0.06	0.013
*RORA*	*7.11 ± 0.13*	*7.21 ± 0.10*	*− 0.10*	*0.018*
*FAM13A*	*7.33 ± 0.14*	*7.43 ± 0.15*	*− 0.10*	*0.049*
UBE2L3*	*7.16 ± 0.16*	*7.28 ± 0.17*	*− 0.12*	*0.035*
ANKRA2	9.10 ± 0.20	9.25 ± 0.17	− 0.15	0.017
THBS1	7.29 ± 0.32	7.62 ± 0.43	− 0.32	0.016

Expression of genes is given as mRNA level. Values are presented as mean ± SD and are log2 transformed. Differences between tertiles were analyzed using the independent samples *t* test. *P* values < 0.05 were considered significant

*Genes differentially expressed between subjects in the highest and lowest n-3 tertile and between subjects in the highest and lowest SFA to PUFA ratio tertile. The genes presented in italics are expressed by more than one probe (shown in Additional file 3: Table S3). The probe with lowest *p*-value is shown in Table 4

**Table 5:** Differentially expressed genes between subjects in the highest and lowest plasma SFA to PUFA ratio tertile

Gene	Highest tertile (n 18)	Lowest tertile (n 18)	Mean difference	*P*
EPHX2*	8.41 ± 0.33	8.18 ± 0.31	0.23	0.036
*FAM117B*	*8.58 ± 0.33*	*8.36 ± 0.09*	*0.22*	*0.012*
CAV1*	7.09 ± 0.27	6.87 ± 0.23	0.22	0.014
SEC24A	7.72 ± 0.26	7.56 ± 0.17	0.16	0.038
UBE2L3**	*7.24 ± 0.18*	*7.09 ± 0.13*	*0.15*	*0.010*
ARV1	8.07 ± 0.16	7.95 ± 0.11	0.12	0.011
NFKB1	11.09 ± 0.10	11.00 ± 0.15	0.09	0.037
SNX13	7.49 ± 0.15	7.40 ± 0.10	0.09	0.045
INSIG2	8.14 ± 0.09	8.07 ± 0.07	0.06	0.025
TMEM188	7.01 ± 0.07	6.95 ± 0.09	0.06	0.034
*ERLIN2*	*6.99 ± 0.06*	*6.93 ± 0.06*	*0.05*	*0.034*
CPS1	6.84 ± 0.07	6.79 ± 0.07	0.05	0.033
TOM1*	*7.04 ± 0.08*	*7.13 ± 0.10*	*− 0.09*	*0.006*
POR	7.75 ± 0.14	7.85 ± 0.11	− 0.10	0.037
GSK3B	8.22 ± 0.15	8.32 ± 0.13	− 0.10	0.021
HMGCR*	8.76 ± 0.19	8.88 ± 0.13	− 0.12	0.015
RBM5	10.81 ± 0.12	10.93 ± 0.15	− 0.12	0.033
*ERGIC3*	*10.54 ± 0.18*	*10.67 ± 0.13*	*− 0.12*	*0.031*
PXN*	8.00 ± 0.14	8.13 ± 0.15	− 0.13	0.014
SCARB1	7.49 ± 0.23	7.62 ± 0.12	− 0.14	0.025
APOBR	8.11 ± 0.18	8.25 ± 0.17	− 0.14	0.038
KLHL8*	*8.96 ± 0.21*	*9.13 ± 0.25*	*− 0.17*	*0.042*
ABCA2	7.41 ± 0.25	7.61 ± 0.30	− 0.20	0.042
PNPLA2	8.28 ± 0.28	8.48 ± 0.30	− 0.20	0.050
STAB1*	8.31 ± 0.50	8.67 ± 0.38	− 0.35	0.023

Expression of genes is given as mRNA level. Values are presented as mean ± SD and are log2 transformed. Differences between tertiles were analyzed using the independent samples *t* test. *P* values < 0.05 were considered significant

*Genes differentially expressed between subjects in the highest and lowest n-6 tertile and between subjects in the highest and lowest SFA to PUFA ratio tertile

**Genes differentially expressed between subjects in the highest and lowest n-3 tertile and between subjects in the highest and lowest SFA to PUFA ratio tertile. The genes presented in italics are expressed by more than one probe (shown in Additional file 4: Table S4). The probe with lowest *p*-value is shown in Table 5

Differentially expressed genes between subjects in the highest and lowest plasma SFA to PUFA ratio tertile included genes encoding proteins involved in cholesterol homeostasis (Table 5). The mRNA levels of *insulin-induced gene 2* (INSIG2), *ER lipid raft associated 2* (ERLIN2), *Caveolin 1* (CAV1), and *COPII subunit SEC24* (SEC24) were significantly higher expressed among subjects in the highest compared to subjects in the lowest plasma SFA to PUFA ratio tertile. The mRNA levels of *scavenger receptor class B member 1* (SCARB1,) *ATP binding-cassette subfamily A member 2* (ABCA2), and *3-hydroxy-3-methylglutaryl-CoA reductase* (HMGCR) were significantly lower expressed among subjects in the highest compared to subjects in the lowest plasma SFA to PUFA ratio tertile. Interestingly, several of the differentially expressed genes associated with plasma SFA to PUFA ratio, including CAV1 and HMGCR, were opposite differentially expressed when comparing subjects in the highest versus the lowest plasma n-6 fatty acid tertile.

In order to examine whether the differentially expressed genes were associated with BMI or serum lipids, correlation analyses were performed (Table 6). BMI was significantly positively correlated to the expression levels of two genes (LACTB and SNX13) and significantly negatively correlated to the expression levels of five genes (ERLIN2, GSK3B, KLHL8, SCARB1, and SELS). The serum level of TG was significantly negatively correlated to the expression levels of five genes (DAGLB, GSK3B, KAT5, KLHL8, and SCARB1). The serum levels of total-C and LDL-C were significantly negatively correlated to the expression levels of two genes (FAM117B and KAT5) and one gene (LRP5), respectively.

**Table 6:** Correlations between subject characteristics and differentially expressed genes associated with plasma n-6 level and/or SFA to PUFA ratio (n 54)

	BMI	TG	Total-C	LDL-C
DAGLB		− 0.31 (*P* 0.020)		
ERLIN2	− 0.31 (*P* 0.021)			
FAM117B			− 0.29 (*P* 0.034)	
GSK3B	− 0.28 (*P* 0.042)	− 0.31 (*P* 0.024)		
KAT5		− 0.33 (*P* 0.016)	− 0.32 (*P* 0.017)	
KLHL8	− 0.29 (*P* 0.034)	− 0.30 (*P* 0.026)		
LACTB	0.31 (*P* 0.023)			
LRP5				− 0.30 (*P* 0.027)
SCARB1	− 0.26 (*P* 0.059)	− 0.27 (*P* 0.045)		
SELS	− 0.32 (*P* 0.033)			
SNX13	0.42 (*P* < 0.01)			

Correlations were analyzed using Pearson’s *r*. *P* values < 0.05 were considered significant

*BMI* body mass index, *TG* triglycerides, *Total-C* total cholesterol, *LDL-C* low-density lipoprotein cholesterol

Even though the percentage number of lymphocytes and monocytes was constant in the three comparisons, we also checked if cell subset-specific genes were differently expressed between the groups (data not shown). We did not observe any significant alterations in the gene expression levels of the B cell-specific gene *CD20*, the T helper cell-specific gene *CD4*, and the monocyte-specific gene *CD14* between the subjects in any of the groups. These findings suggest that there is no indication that there is a difference in B lymphocyte/monocyte ratio in the comparisons. However, we observed a significant lower mRNA level of the cytotoxic T lymphocyte-specific gene *CD8A* in the high versus low plasma n-6 level group, and significant higher mRNA levels of *CD8A* and *CD8B* in the high versus low plasma SFA to PUFA ratio group. This may indicate that plasma fatty acids may influence the T lymphocyte/monocyte ratio.

## Discussion

In this explorative study, we investigated the potential relation of plasma n-6 and n-3 fatty acid levels, and plasma SFA to PUFA ratio, to PBMC gene expression related to lipid metabolism in healthy subjects. The plasma SFA to PUFA ratio was associated with the highest number of differentially expressed genes, followed by plasma n-6 and n-3 fatty acid level. In particular, genes involved in cholesterol metabolism were differentially expressed.

PBMCs have previously been shown to reflect hepatic lipid metabolism during fasting [23]. In addition, genes related to lipid metabolism have been shown to be differentially expressed in PBMCs after acute meal studies with different fat qualities [26, 27]. In the present study, we used a targeted approach to investigate whether 285 genes encoding proteins related to cholesterol and TG metabolism were differentially expressed depending on plasma fatty acid levels. Among the 285 genes, a total of 161 genes were expressed in the PBMCs. Interestingly, it seems like the plasma SFA to PUFA ratio is a stronger determinant than the plasma levels of n-6 and n-3 fatty acids alone regarding the potential of influencing gene expression levels in PBMCs. PBMCs may therefore function as a good model system to get a better understanding of how genes involved in lipid metabolism are regulated by the plasma fatty acid levels and in particular the SFA to PUFA ratio.

Genes involved in the regulation of cholesterol homeostasis were differentially expressed among subjects with high compared to low plasma SFA to PUFA ratio. ABCA2 encodes a member of the ATP-binding cassette (ABC) transporters, a subfamily of transporters that have been functionally linked to intracellular lipid transport [38]. The mRNA level of ABCA2 was significantly lower among subjects with high compared to low plasma SFA to PUFA ratio. It has been demonstrated that ABCA2 positively regulates low-density lipoprotein receptor (LDLR) mRNA expression and negatively regulates cholesterol esterification in hamster ovary cells [39]. In addition, it has been shown that overexpression of ABCA2 in neuroblastoma cells results in decreased cellular cholesterol levels [40]. Thus, our findings suggest that a lower ABCA2 expression level may lead to lower uptake and lower synthesis of cholesterol.

Because most cells in the periphery of the body do not express pathways for catabolizing cholesterol, efflux of cholesterol is critical for maintaining cholesterol homeostasis. SCARB1 and CAV1 are genes encoding proteins involved in cholesterol efflux. The mRNA level of CAV1 was significantly higher among subjects with high compared to low plasma SFA to PUFA ratio. It has been shown that CAV1 regulates the ATP-binding cassette subfamily G member 1 (ABCG1)-mediated efflux of cholesterol, probably by regulating ABCG1 trafficking to the cell surface [41], and a potential increase in CAV1 will subsequently lead to increased cholesterol efflux through ABCG1. Interestingly, there was also a significantly lower mRNA level of CAV1 among subjects with high compared to low plasma n-6 fatty acid level. As n-6 fatty acids are the majority of total plasma PUFAs, this finding may suggest that SFAs and PUFAs may exert different effects on cholesterol efflux pathways. We did not observe a significant difference in the mRNA level of ABCG1 among subjects with high compared to low plasma SFA to PUFA ratio. However, there was a significantly lower mRNA level of SCARB1, which encodes another plasma membrane receptor mediating cholesterol transfer to and from HDL. In contrast to ABCG1, which mediate cholesterol efflux via active transport, SCARB1 mediates cholesterol transport to and from HDL via passive facilitated diffusion [42]. In cholesterol-loaded mouse macrophages incubated with diluted human serum, it has been shown that cholesterol efflux is mainly mediated by active transport [43]. We therefore speculate that there may be a higher efflux of cholesterol via active transport than by passive transport in response to a high intracellular cholesterol load, which may explain the lower expression level of SCARB1 in subjects with high compared to low plasma SFA to PUFA ratio.

In addition, there was a significantly lower mRNA level of HMGCR among subjects with high compared to low SFA to PUFA ratio, and the mRNA level of HMGCR was significantly higher among subjects with high compared to low plasma n-6 PUFA level. HMGCR encodes the rate limiting step in the cholesterol biosynthesis pathway, and a lower expression of this gene will subsequently lead to reduced synthesis of cholesterol. The finding in the present study is in line with a recent postprandial study investigating the effects of SFA in lean and obese subjects [27]. It is interesting that although the abovementioned study investigated the acute effects of a high fat meal, we were able to observe a similar change in the mRNA level of HMGCR among subjects with higher plasma SFA to PUFA ratio compared to subjects with lower plasma SFA to PUFA ratio. The transcription of HMGCR is regulated by sterol regulatory element-binding proteins (SREBPs) [44]. SREBP2 stimulates the transcription of genes involved in cholesterol biosynthesis and uptake [45]. We did not observe a significant difference in the mRNA level of SREBP2 among subjects with high compared to low SFA to PUFA ratio, nor did we observe a significant difference in the expression level of the *LDL receptor* (LDLR) which is also regulated by SREBP2. However, several genes involved in the proteolytic regulation of SREBP2, including INSIG2 and ERLIN2, were significantly higher expressed among subjects with high compared to low SFA to PUFA ratio. INSIG2 binds to cholesterol-loaded sterol binding proteins (SCAPs) in the endoplasmic reticulum (ER) and prevents the movement of the SCAP-SREBP complex to the Golgi apparatus for further processing and eventually transcription of HMGCR and other SREBP target genes. In addition, INSIG proteins play an important role in oxysterol-regulated cleavage of SREBPs [46]. ERLIN2 encodes a cholesterol-sensing protein which has been suggested to stabilize the SREBP-SCAP-INSIG complex in the ER [47]. This gene was also significantly negatively correlated with BMI, thereby implicating that BMI impact on intracellular cholesterol levels. Thus, a higher mRNA level of INSIG2 and ERLIN2 is in line with the lower mRNA level of HMGCR observed in the present study. Additionally, we observed a higher mRNA level of SEC24 among subjects with high compared to low plasma SFA to PUFA ratio. SEC24 is a component of the COP11-coated vesicles, which transport the SREBP-SCAP complex to the Golgi. When ER cholesterol rises above a threshold of total lipids, the cholesterol binds to SCAP, which triggers a conformational change in the protein that occludes the binding of the COPII proteins [48]. We speculate that a higher expression level of SEC24 may be a response to a higher intracellular cholesterol level among subjects with high compared to low plasma SFA to PUFA ratio.

Although we did not observe differences in serum cholesterol levels among subjects with high compared to low plasma SFA to PUFA ratio, the differentially expressed genes observed in the present study may reflect an intracellular status of excess cholesterol. Changes in the expression of genes occur prior to changes at protein level, and these findings may therefore reflect early changes related to diet. The question remains how the abovementioned genes are potentially regulated by changes in the plasma SFA to PUFA ratio. It is well known that dietary intake of PUFA, in particular of LA, AA, EPA, and DHA, correlate with their respective percentages in plasma total fatty acids [14]. However, the total plasma fatty acid profile may not reflect the total dietary intake of fat as the fatty acid composition in different lipid fractions differs depending on the fat intake [49]. However, we have shown recently in a dietary intervention study where SFA were replaced with PUFA that the total plasma fatty acid profile reflected dietary fat intake changes [8]. It is clear that fatty acids have the ability to regulate the expression of genes involved in lipid metabolism. PUFAs have been shown to decrease nuclear SREBP-1 protein levels in part by inhibition of the interaction of oxysterols with LXR; however, this mechanism does not seem to affect SREBP2 [50]. The findings in the present study may therefore be explained by the plasma levels of SFAs. Recent findings suggest that SFAs may decrease SREBP activity directly, but the exact mechanisms whereby SFAs may exert their effects on SREBP and its downstream targets remain to be established [19].

The present study has several strengths. Gene expression profiling in PBMCs has been shown to be more sensitive to dietary changes than the traditional biochemical parameters in the circulation, and we specifically choose genes involved in biological processes related to TG and cholesterol metabolism. In the present study, we have compared the mRNA levels with plasma levels of n-3, n-6, and the SFA to PUFA ratio. Since intake of the n-3 and the n-6 fatty acids are reflected in the plasma total fatty acid composition, our data indicate that intake of these fatty acids may cause differences in gene expression. The major limitation of the present study is the limited number of subjects. Due to the small number of subjects, the subjects were separated into groups based on tertiles, which may have had an impact on the number of significantly differentially expressed genes observed between the highest and lowest tertiles. However, since we used end of study samples from a fish oil intervention, we could at least expect a larger variation in plasma n-3 fatty acid level among the subjects. Another limitation is that that there may be a different T lymphocyte/monocyte ratio in the n-6 tertile groups and the SFA to PUFA tertile groups. Whether the mRNA levels of CD8A and CD8B are linked to the plasma level of the fatty acids or by the change in the number of subset cells cannot be determined by this cross-sectional study. Another limitation is the lack of PBMC material to perform protein measurements to validate our mRNA results. Since this was an explorative study, we did not adjust for multiple testing. Although no causal relationship can be made due to the cross-sectional design of the study, the current study shows that the plasma fatty acid levels can influence the PBMC expression of genes involved in lipid metabolism.

## Conclusion

In conclusion, the main findings in the present study were that PBMCs express genes involved in hepatic lipid metabolism and that the expression of several of the genes was influenced by plasma fatty acid levels. This finding supports the use of PBMCs as a model system for investigating the role dietary n-3 and n-6 fatty acids on gene expression related to lipid metabolism. The plasma SFA to PUFA ratio seems to be more important than the plasma n-6 and n-3 fatty acid level alone with regard to influencing mRNA levels. In particular, genes involved in cholesterol homeostasis were significantly differently expressed among subjects with high compared to low plasma SFA to PUFA ratio. This may reflect an intracellular status of excess cholesterol among subjects with high plasma SFA to PUFA ratio. The current findings should be further studied in experimental studies and tested in well-controlled human dietary intervention studies.

## Additional files

Additional file 1: Selection of genes related to triglyceride- and cholesterol metabolism (285 genes). Genes expressed in peripheral blood mononuclear cells are in bold (161 genes). (DOCX 31 kb)

Additional file 2: Differentially expressed genes associated with plasma n-6 level and expressed by more than one probe. (DOCX 19 kb)

Additional file 3: Differentially expressed genes associated with plasma n-3 level and expressed by more than one probe. (DOCX 16 kb)

Additional file 4: Differentially expressed genes associated with plasma SFA to PUFA ratio and expressed by more than one probe. (DOCX 17 kb)
