# Correction to: Plasma fatty acid levels and gene expression related to lipid metabolism in peripheral blood mononuclear cells: a cross-sectional study in healthy subjects

**DOI:** 10.1186/s12263-018-0604-8

**Published:** 2018-07-05

**Authors:** Sunniva V. Larsen, Kirsten B. Holven, Inger Ottestad, Kine N. Dagsland, Mari C. W. Myhrstad, Stine M. Ulven

**Affiliations:** 1 Department of Nutrition, Institute for Basic Medical Sciences, University of Oslo, P.O. Box 1046, Blindern, 0317 Oslo, Norway; 2 Norwegian National Advisory Unit on Familial Hypercholesterolemia, Department of Endocrinology, Morbid Obesity and Preventive Medicine, Oslo University Hospital, P.O. Box 4950, Nydalen, 0424 Oslo, Norway; 3 Department of Health, Nutrition and Management, Faculty of Health Sciences, Oslo and Akershus University College of Applied Sciences, P.O. Box 4, St. Olavs plass, 0130 Oslo, Norway

## Correction

Unfortunately, after publication of this article [1], it was noticed that some corrections to Table 2 were not carried out. The corrected Table 2 can be seen below and the original article has also been updated to reflect this.

**Table 2:** Characteristics and plasma fatty acid profile of subjects in the highest and lowest tertiles

	n-6 level			n-3 level			SFA/PUFA ratio		
	Highest tertile	Lowest tertile	*P*	Highest tertile	Lowest tertile	*P*	Highest tertile	Lowest tertile	*P*
	(n 18)	(n 18)		(n 18)	(n 18)		(n 18)	(n 18)	
Male/female	5/13	5/13		3/15	5/13		5/13	6/12	
Age (years)	25 (23–33)	25 (21–29)	0.31	25 (21–31)	24 (21–28)	0.80	25 (22–29)	28 (23.0–32.8)	0.38
BMI (kg/m2)	22.5 ± 2.9	22.6 ± 2.6	0.94	22.2 ± 2	22.7 ± 3.3	0.57	23.3 ± 2.7	22.6 ± 2.6	0.41
Total-C (mmol/l)	4.9 ± 0.9	4.7 ± 1.1	0.65	4.9 ± 1	4.9 ± 1	0.96	4.7 ± 0.8	5.0 ± 0.9	0.25
LDL-C (mmol/l)	2.7 ± 0.8	2.6 ± 0.9	0.79	2.6 ± 0.9	2.8 ± 0.6	0.71	2.5 ± 0.6	2.8 ± 0.9	0.16
HDL-C (mmol/l)	1.6 ± 0.4	1.4 ± 0.3	0.15	1.6 ± 0.3	1.4 ± 0.4	0.28	1.5 ± 0.4	1.6 ± 0.3	0.74
TG (mmol/l)	0.8 (0.6–0.9)	1.2 (0.9–1.7)	*0.01*	0.8 (0.6–1.1)	1.0 (0.7–1.2)	0.30	1.1 (0.8–1.6)	0.8 (0.6–0.9)	*0.01*
Lymphocytes (%)	38.4 ± 6.8	43.0 ± 8.5	0.08	42.2 ± 10.0	37.6 ± 4	0.09	42.1 ± 8.7	37.4 ± 8.4	0.11
Monocytes (%)	8.5 ± 1.8	8.1 ± 1.9	0.49	8.1 ± 1.7	8.6 ± 2.3	0.42	8.8 ± 2.2	8.48 ± 2.0	0.71
Plasma level of fatty (wt%)									
Total SFA	29.4 (28.8–30.5)	32.1 (31.1–33.2)	*< 0.01*	30.7 (29.3–32.3)	30.8 (29.8–31.8)	0.78	32.6 (31.5–33.2)	29.1 (28.7–29.5)	*< 0.01*
Myristic acid (14:0)	0.6 (0.6–1.0)	1.0 (0.8–1.2)	*< 0.01*	0.8 (0.6–1.1)	0.9 (0.7–1.1)	0.57	1.1 (0.9–1.2)	0.6 (0.6–0.9)	*< 0.01*
Palmitic acid (16:0)	20.5 (20.2–21.6)	23.4 (21.4–24.2)	*< 0.01*	20.9 (20.4–23.7)	21.5 (20.9–22.6)	0.39	23.9 (21.8–24.5)	20.4 (20.2–20.7)	*< 0.01*
Stearic acid (18:0)	8.1 ± 0.7	8.1 ± 1.1	0.86	8.2 ± 1.1	8.0 ± 1.0	0.65	8.0 ± 1.1	7.7 ± 0.8	0.37
Total n-6	38.6 ± 1.3	30.3 ± 2.9	*< 0.01*	31.0 ± 4.0	31.5 ± 4.5	0.74	30.8 ± 3.4	38.0 ± 2.0	*< 0.01*
LA (18:2n-6)	32.3 ± 1.6	24.9 ± 2.5	*< 0.01*	27.8 ± 3.8	29.2 ± 4.1	0.29	24.8 ± 2.5	31.8 ± 2.1	*< 0.01*
AA (20:4n-6)	6.2 ± 1.1	5.4 ± 0.9	*0.02*	5.8 ± 0.7	6.2 ± 1.2	0.32	5.9 ± 1.3	6.2 ± 1. 1	0.58
Total n-3	5.8 ± 2.5	7.0 ± 2.3	0.12	9.0 ± 0.9	3.6 ± 0.49	*< 0.01*	6.1 ± 2.5	6.8 ± 2.4	0.44
ALA (18:3n- 3)	0.5 (0.5–0.6)	0.5 (0.4–0.6)	0.46	0.5 (0.5–0.6)	0.5 (0.5–0.6)	0.79	0.5 (0.5–0.6)	0.5 (0.5–0.5)	0.95
EPA (20:5n-3)	1.7 (0.6–2.3)	2.4 (1.8–2.9)	0.14	3.2 (2.8–3.7)	0.6 (0.5–0.6)	*< 0.01*	2.0 (0.7–2.6)	2.2 (1.3–3.2)	0.46
DPA (22:5n-3)	0.6 ± 0.1	0.7 ± 0.2	0.26	0.8 ± 0.1	0.5 ± 0.1	*< 0.01*	0.6 ± 0.2	0.6 ± 0.1	0.80
DHA (22:6n-3)	3.0 (1.9–4.1)	4.0 (3.1–4.1)	0.14	4.3 (4.1–4.7)	1.9 (1.8–2.1)	*< 0.01*	3.5 (2.0–4.1)	3.3 (2.8–4.3)	0.48
Total PUFA	43.8 (42.1–47.3)	38.6 (35.8–39.7)	*< 0.01*	42.2 (39.5–46.7)	40.4 (37.7–41.5)	*0.05*	37.8 (35.8–39.1)	44.6 (43.1–47.3)	*< 0.01*
SFA to PUFA ratio	0.7 (0.6–0.7)	0.8 (0.8–0.9)	*< 0.01*	0.7 (0.6–0.8)	0.8 (0.7–0.8)	0.13	0.9 (0.8–0.9)	0.7 (0.6–0.7)	*< 0.01*

Lymphocytes and monocytes are given as percentage of total white cell count. Differences between tertiles were analyzed using the independent samples *t* test when normally distributed or the Mann-Whitney *U* test when not normally distributed. *P* values < 0.05 were considered significant. Values are presented as mean ± SD or medians and 25th–75th percentile

*BMI* body mass index, *Total-C* total cholesterol, *LDL-C* low-density lipoprotein cholesterol, *HDL-C* high-density lipoprotein cholesterol, *TG* triglycerides, *SFA* saturated fatty acid, *LA* linoleic acid, *AA* arachidonic acid, *ALA* alpha-linolenic acid, *EPA* eicosapentaenoic acid, *DPA* docosapentaenoic acid, *DHA* docosahexaenoic acid, *PUFA* polyunsaturated fatty acid
