# Supplementary material for: Plasma fatty acid levels and gene expression related to lipid metabolism in peripheral blood mononuclear cells: a cross-sectional study in healthy subjects
Source: Genes Nutr. 2018 Apr 10;13:9. doi: 10.1186/s12263-018-0600-z (PMC5892037; doi:10.1186/s12263-018-0600-z)
Supplement: Supplementary file 1 — Selection of genes related to triglyceride- and cholesterol metabolism (285 genes). Genes expressed in peripheral blood mononuclear cells are in bold (161 genes). (DOCX 31 kb) [file 12263_2018_600_MOESM1_ESM.docx]

**Table S1.** Selection of genes related to triglyceride- and cholesterol metabolism (285 genes). Genes expressed in peripheral blood mononuclear cells are in bold (161 genes).

|  |  |
| --- | --- |
| Official gene symbol | Official gene name |
| AACS | acetoacetyl-CoA synthetase |
| ABCA1 | ATP-binding cassette sub-family A member 1 |
| ABCA12 | ATP-binding cassette sub-family A member 12 |
| ABCA2 | ATP-binding cassette sub-family A member 2 |
| ABCA5 | ATP-binding cassette sub-family A member 5 |
| ABCA7 | ATP-binding cassette sub-family A member 7 |
| ABCA8 | ATP binding cassette subfamily A member 8 |
| ABCB11 | ATP binding cassette subfamily B member 11 |
| ABCB4 | ATP-binding cassette sub-family B member 4 |
| ABCG1 | ATP-binding cassette sub-family G member 1 |
| ABCG4 | ATP-binding cassette sub-family G member 4 |
| ABCG5 | ATP-binding cassette sub-family G member 5 |
| ABCG8 | ATP-binding cassette sub-family G member 8 |
| ABHD5 | abhydrolase domain containing 5 |
| ABO | ABO, alpha 1-3-N-acetylgalactosaminyltransferase and alpha 1-3-galactosyltransferase |
| ACAD11 | acyl-CoA dehydrogenase family member 11 |
| ACADL | acyl-CoA dehydrogenase long chain |
| ACADVL | acyl-CoA dehydrogenase very long chain |
| ACOX1 | acyl-CoA oxidase 1 |
| ACSM1 | acyl-CoA synthetase medium chain family member 1 |
| ACSM2A | acyl-CoA synthetase medium chain family member 2A |
| ACSM3 | acyl-CoA synthetase medium chain family member 3 |
| ADH5 | alcohol dehydrogenase 5 (class III), chi polypeptide |
| ADIPOQ | adiponectin, C1Q and collagen domain containing |
| AGT | angiotensinogen (serpin peptidase inhibitor, clade A, member 8) |
| AGTR1 | angiotensin II receptor type 1 |
| AKR1C1 | aldo-keto reductase family 1 member C1 |
| AKR1C4 | aldo-keto reductase family 1 member C4 |
| AMPD3 | adenosine monophosphate deaminase 3 |
| ANGPTL1 | angiopoietin like 1 |
| ANGPTL3 | angiopoietin like 3 |
| ANGPTL4 | angiopoietin like 4 |
| ANGPTL8 | angiopoietin like 8 |
| ANKRA2 | ankyrin repeat family A member 2 |
| APOA1 | apolipoprotein A-1 |
| APOA2 | apolipoprotein A-2 |
| APOA4 | apolipoprotein A-4 |
| APOA5 | apolipoprotein A-5 |
| APOB | apolipoprotein B |
| APOBR | apolipoprotein B receptor |
| APOC1 | apolipoprotein C1 |
| APOC2 | apolipoprotein C2I |
| APOC3 | apolipoprotein C3 |
| APOC4 | apolipoprotein C4 |
| APOE | apolipoprotein E |
| APOF | apolipoprotein F |
| APOH | apolipoprotein H |
| APOL1 | apolipoprotin L 1 |
| APOM | apolipoprotein M |
| APOO | apolipoprotein O |
| ARL15 | ADP ribosylation factor like GTPase 15 |
| ARV1 | sterol homeostasis protein ARV1 |
| ASAP3 | ArfGAP with SH3 domain, ankyrin repeat and PH domain 3 |
| ATG7 | autophagy related 7 |
| BRAP | BRCA1 associated protein |
| BRCA2 | BRCA2, DNA repair associated |
| C10orf112 | chromosome 10 open reading frame 112 |
| C19orf80 | chromosome 19 open reading frame 80 |
| C1QTNF3 | C1q and tumor necrosis factor related protein 3 |
| C3 | complement component 3 |
| C6orf106 | chromosome 6 open reading frame 106 |
| CAPN3 | calpain 3 |
| CAV1 | caveolin 1 |
| CAV3 | caveolin 3 |
| CD24 | CD24 molecule |
| CD36 | CD36 molecule |
| CDH13 | cadherin 13 |
| CETD2 | SET domain containing 2 |
| CETP | cholesteryl ester transfer protein |
| CIDEA | cell death-inducing DFFA-like effector a |
| CILP2 | cartilage intermediate layer protein 2 |
| CITED2 | Cbp/p300 interacting transactivator with Glu/Asp rich carboxy-terminal domain 2 |
| CLU | clusterin |
| CMIP | c-Maf inducing protein |
| CMTM5 | CKLF like MARVEL transmembrane domain containing 5 |
| COBLL1 | cordon-bleu WH2 repeat protein like 1 |
| COLEC12 | collectin sub-family member 12 |
| CPS1 | carbamoyl-phosphate synthase 1 |
| CRP | C-reactive protein |
| CSNK1G3 | casein kinase 1 gamma 3 |
| CTDNEP1 | CTD nuclear envelope phosphatase 1 |
| CTF1 | cardiotrophin 1 |
| CYP26A1 | cytochrome P450 family 26 subfamily A member 1 |
| CYP7A1 | cytochrome P450 family 7 subfamily A member 1 |
| DAGLB | diacylglycerol lipase beta |
| DGAT2 | diacylglycerol O-acyltransferase 2 |
| DHCR7 | 7-dehydrocholesterol reductase |
| DLG4 | discs large MAGUK scaffold protein 4 |
| DNAH11 | dynein axonemal heavy chain 11 |
| EGF | epidermal growth factor |
| EHBP1 | EH domain binding protein 1 |
| EHD1 | EH-domain containing 1 |
| EPHX2 | epoxide hydrolase 2, cytoplasmic |
| ERGIC3 | ERGIC and golgi 3 |
| ERLIN1 | ER lipid raft associated 1 |
| ERLIN2 | ER lipid raft associated 2 |
| EVI5 | ecotropic viral integration site 5 |
| FABP3 | fatty acid binding protein 3 |
| FABP4 | fatty acid binding protein 4 |
| FADS1 | fatty acid desaturase 1 |
| FADS2 | fatty acid desaturase 2 |
| FADS3 | fatty acid desaturase 3 |
| FAM117B | family with sequence similarity 117 member B |
| FAM13A | family with sequence similarity 13 member A |
| FBXW7 | F-box and WD repeat domain containing 7 |
| FGF1 | fibroblast growth factor 1 |
| FGFR4 | fibroblast growth factor receptor 4 |
| FITM1 | fat storage-inducing transmembrane protein 1 |
| FITM2 | fat storage-inducing transmembrane protein 2 |
| FN1 | fibronectin 1 |
| FRK | fyn related Src family tyrosine kinase |
| FRMD5 | FERM domain containing 5 |
| FTO | FTO, alpha-ketoglutarate dependent dioxygenase |
| G6PC | glucose-6-phosphatase catalytic subunit |
| GALNT2 | polypeptide N-acetylgalactosaminyltransferase 2 |
| GCKR | glucokinase regulator |
| GIP | gastric inhibitory polypeptide |
| GPAM | glycerol-3-phosphate acyltransferase |
| GPIHBP1 | glycosylphosphatidylinositol anchored high density lipoprotein binding protein 1 |
| GPLD1 | glycosylphosphatidylinositol specific phospholipase D1 |
| GPR 146 | G protein-coupled receptor 146 |
| GSK3B | glycogen synthase kinase 3 beta |
| HAS1 | hyaluronan synthase 1 |
| HBS1L | HBS1 like translational GTPase |
| HDLBP | high density lipoprotein binding protein |
| Hfe | hemochromatosis |
| HMGCR | 3-hydroxy-3-methylglutaryl-CoA reductase |
| HMGCS1 | 3-hydroxy-3-methylglutaryl-CoA synthase 1 (soluble) |
| HNF1A | hepatocyte nuclear factor 1 alpha |
| HNF4A | hepatocyte nuclear factor 4 alpha |
| HPR | haptoglobin-related protein |
| IKZF1 | IKAROS family zinc finger 1 |
| INHBA | inhibin beta A subunit |
| INSIG2 | insulin induced gene 2 |
| INSR | insulin receptor |
| IRF2BP2 | interferon regulatory factor 2 binding protein 2 |
| IRS1 | insulin receptor substrate 1 |
| JMJD1C | jumonji domain containing 1C |
| KAT5 | lysine acetyltransferase 5 |
| KCNK17 | potassium two pore domain channel subfamily K member 17 |
| KLHL8 | kelch like family member 14 |
| LACTB | lactamase beta |
| LAMTOR1 | late endosomal/lysosomal adaptor, MAPK and MTOR activator 1 |
| LCAT | lecithin-cholesterol acyltransferase |
| LDLR | low density lipoprotein receptor |
| LDLRAP1 | low density lipoprotein receptor adaptor protein 1 |
| LEP | leptin |
| LILRA3 | leukocyte immunoglobulin like receptor A3 |
| LIPC | Lipase C, hepatic type |
| LIPG | Lipase G, endothelial type |
| LMF1 | lipase maturation factor 1 |
| LPA | lipoprotein(a) |
| LPL | lipoprotein lipase |
| LRP1 | low density lipoprotein receptor related protein 1 |
| LRP4 | low density lipoprotein receptor related protein 4 |
| LRP5 | low density lipoprotein receptor related protein 5 |
| LRP6 | low density lipoprotein receptor related protein 6 |
| LRP8 | low density lipoprotein receptor related protein 8 |
| LRPAP1 | LDL receptor related protein associated protein 1 |
| LSR | lipolysis stimulated lipoprotein receptor |
| MAFB | MAF bZIP transcription factor B |
| MALL | mal, T-cell differentiation protein like |
| MAMSTR | MEF2 activating motif and SAP domain containing transcriptional regulator |
| MAP3K1 | mitogen-activated protein kinase kinase kinase 1 |
| MC4R | melanocortin 4 receptor |
| MED13 | mediator complex subunit 13 |
| MET | MET proto-oncogene, receptor tyrosine kinase |
| MIA2 | melanoma inhibitory activity 2 |
| MIR148A | microRNA 148a |
| MLC1 | megalencephalic leukoencephalopathy with subcortical cysts 1 |
| MLXIPL | MLX interacting protein-like |
| MOSC1 | mitochondrial amidoxime reducing component 1 |
| MPO | myeloperoxidase |
| MPP3 | membrane palmitoylated protein 3 |
| MSL2L1 | MSL complex subunit 2 |
| MSR1 | macrophage scavenger receptor 1 |
| MTMR3 | myotubularin related protein 3 |
| MVK | mevalonate kinase |
| MYLIP | myosin regulatory light chain interacting protein |
| NAT2 | N-acetyltransferase 2 |
| NFKB1 | nuclear factor kappa B subunit 1 |
| NFKBIA | NFKB inhibitor alpha |
| NPC1 | NPC intracellular cholesterol transporter 1 |
| NPC1L1 | NPC1 like intracellular cholesterol transporter 1 |
| NPC2 | NPC intracellular cholesterol transporter 2 |
| NR1D1 | nuclear receptor subfamily 1 group D member 1 |
| NR1H2 | nuclear receptor subfamily 1 group H member 2 |
| NR1H3 | nuclear receptor subfamily 1 group H member 3 |
| NR1H4 | nuclear receptor subfamily 1 group H member 4 |
| NR5A2 | nuclear receptor subfamily 5 group A member 2 |
| NUS1 | NUS1 dehydrodolichyl diphosphate synthase subunit |
| NYNRIN | NYN domain and retroviral integrase containing |
| OR4C46 | olfactory receptor family 4 subfamily C member 46 |
| OSBPL11 | oxysterol binding protein like 11 |
| OSBPL7 | oxysterol binding protein like 7 |
| OSBPL8 | oxysterol binding protein like 8 |
| PABPC4 | poly(A) binding protein cytoplasmic 4 |
| PCSK9 | proprotein convertase subtilisin/kexin type 9 |
| PCYOX1 | prenylcysteine oxidase 1 |
| PDE3A | phosphodiesterase 3A |
| PEPD | peptidase D |
| PGS1 | phosphatidylglycerophosphate synthase 1 |
| PHLDB1 | pleckstrin homology like domain family B member 1 |
| PINX1 | PIN2/TERF1 interacting telomerase inhibitor 1 |
| PLA2G10 | phospholipase A2 group X |
| PLA2G2A | phospholipase A2 group IIA |
| PLA2G6 | phospholipase A2 group VI |
| PLA2G7 | phospholipase A2 group VII |
| PLEC1 | plectin |
| PLIN5 | perilipin 5 |
| PLSCR3 | phospholipid scramblase 3 |
| PLTP | phospholipid transfer protein |
| PNLIP | pancreatic lipase |
| PNPLA2 | patatin like phospholipase domain containing 2 |
| PON1 | paraoxonase 1 |
| POR | cytochrome p450 oxidoreductase |
| PPARA | peroxisome proliferator activated receptor alpha |
| PPARG | peroxisome proliferator activated receptor gamma |
| PPP1R3B | protein phosphatase 1 regulatory subunit 3B |
| PRKAA1 | protein kinase AMP-activated catalytic subunit alpha 1 |
| PTCH1 | patched 1 |
| PTCHD2 | patched domain containing 2 |
| PXN | paxillin |
| RAB3GAP1 | RAB3 GTPase activating protein catalytic subunit 1 |
| RAF1 | Raf-1 proto-oncogene, serine/threonine kinase |
| RBM5 | RNA binding motif protein 5 |
| RGN | regucalcin |
| RORA | RAR-related orphan receptor A |
| RSPO3 | R-spondin 3 [Homo sapiens |
| SAA1 | serum amyloid A1 |
| SAA2 | serum amyloid A2 |
| SAA4 | serum amyloid A4, constitutive |
| SBNO1 | strawberry notch homolog 1 |
| SCAP | SREBF chaperone |
| SCARB1 | scavenger receptor class B member 1 |
| SCARF1 | scavenger receptor class F member 1 |
| SCP2 | sterol carrier protein 2 |
| SEC14L2 | SEC14 like lipid binding 2 |
| SEC24A | SEC24 homolog A, COPII coat complex component |
| SELS | selenoprotein S |
| SERPINA12 | serpin family A member 12 |
| SESN2 | sestrin 2 |
| SHH | sonic hedgehog |
| SIK1 | salt-inducible kinase 1 |
| SIRT1 | sirtuin 1 |
| SLC39A8 | solute carrier family 39 member 8 |
| SMIM20 | small integral membrane protein 20 |
| SMO | smoothened, frizzled class receptor |
| SNX13 | sorting nexin 13 |
| SNX5 | sorting nexin 5 |
| SOAT1 | sterol O-acyltransferase 1 |
| SOAT2 | sterol O-acyltransferase 2 |
| SOD1 | superoxide dismutase 1 |
| SORL1 | sortilin related receptor 1 |
| SORT1 | sortilin 1 |
| SOX17 | SRY-box 17 |
| SPTLC3 | serine palmitoyltransferase long chain base subunit 3 |
| SPTY2D1 | SPT2 chromatin protein domain containing 1 |
| SREBF1 | sterol regulatory element binding transcription factor 1 |
| SREBF2 | sterol regulatory element binding transcription factor 2 |
| ST3GAL4 | ST3 beta-galactoside alpha-2,3-sialyltransferase 4 |
| STAB1 | stabilin 1 |
| STAB2 | stabilin 2 |
| STARD3 | StAR related lipid transfer domain containing 3 |
| THBS1 | thrombospondin 1 |
| THRSP | thyroid hormone responsive |
| TIMD4 | T-cell immunoglobulin and mucin domain containing 4 |
| TMEM176A | transmembrane protein 176A |
| TMEM188 | transmembrane protein 188 |
| TMEM97 | transmembrane protein 97 |
| TOM1 | target of myb1 membrane trafficking protein |
| TOP1 | DNA topoisomerase I |
| TRIB1 | tribbles pseudokinase 1 |
| TRPS1 | transcriptional repressor GATA binding 1 |
| TSPO | translocator protein |
| TTC39B | tetratricopeptide repeat domain 39B |
| TYW1B | tRNA-yW synthesizing protein 1 homolog B |
| UBASH3B | ubiquitin associated and SH3 domain containing B |
| UBE2L3 | ubiquitin conjugating enzyme E2 L3 |
| UGT1A1 | UDP glucuronosyltransferase family 1 member A1 |
| VEGFA | vascular endothelial growth factor A |
| VLDLR | very low density lipoprotein receptor |
| XBP1 | X-box binding protein 1 |
| ZNF648 | zinc finger protein 648 |
| ZNF664 | zinc finger protein 664 |
