# Supplementary material for: Plasma fatty acid levels and gene expression related to lipid metabolism in peripheral blood mononuclear cells: a cross-sectional study in healthy subjects
Source: Genes Nutr. 2018 Apr 10;13:9. doi: 10.1186/s12263-018-0600-z (PMC5892037; doi:10.1186/s12263-018-0600-z)
Supplement: Supplementary file 2 — Differentially expressed genes associated with plasma n-6 level and expressed by more than one probe. (DOCX 19 kb) [file 12263_2018_600_MOESM2_ESM.docx]

Table S2. Differentially expressed genes associated with plasma n-6 level and expressed by more than one probe

| Gene | Highest tertile (n 18) | | Lowest tertile (n 18) | | Mean difference | P |
| --- | --- | --- | --- | --- | --- | --- |
| SORL1_1 | **10.25** | **0.27** | **10.06** | **0.27** | **0.19** | **0.039** |
| SORL1_2 | 10.37 | 0.18 | 10.33 | 0.23 | 0.04 | 0.585 |
| KLHL8_1 | 8.12 | 0.27 | 8.18 | 0.14 | -0.07 | 0.373 |
| KLHL8_2 | **9.13** | **0.25** | **8.94** | **0.19** | **0.18** | **0.020** |
| TOM1_1 | **9.78** | **0.24** | **9.62** | **0.20** | **0.16** | **0.037** |
| TOM1_2 | 7.12 | 0.09 | 7.05 | 0.10 | 0.07 | 0.039 |
| KAT5_1 | 7.27 | 0.09 | 7.21 | 0.10 | 0.06 | 0.055 |
| KAT5_2 | **7.45** | **0.09** | **7.39** | **0.09** | **0.06** | **0.047** |
| LACTB_1 | 7.57 | 0.27 | 7.61 | 0.20 | -0.05 | 0.552 |
| LACTB_2 | 8.76 | 0.35 | 8.85 | 0.37 | -0.09 | 0.441 |
| LACTB_3 | **7.15** | **0.12** | **7.24** | **0.12** | **-0.09** | **0.027** |
| SELS_1 | 7.01 | 0.05 | 7.03 | 0.07 | -0.02 | 0.265 |
| SELS_2 | **9.66** | **0.12** | **9.83** | **0.27** | **-0.18** | **0.020** |
| SELS_3 | 8.01 | 0.17 | 8.12 | 0.29 | -0.12 | 0.150 |
| XBP1_1 | **10.63** | **0.23** | **10.85** | **0.37** | **-0.22** | **0.042** |
| XBP1_2 | 10.81 | 0.19 | 10.95 | 0.36 | -0.14 | 0.164 |

Expression of genes is given as mRNA level. Values are presented as mean ± SD and are log2 transformed. Differences between tertiles were analysed using the Independent Samples t-test. P-values <0.05 were considered significant. The probes in bold are shown in Table 3.
