# Supplementary material for: Plasma fatty acid levels and gene expression related to lipid metabolism in peripheral blood mononuclear cells: a cross-sectional study in healthy subjects
Source: Genes Nutr. 2018 Apr 10;13:9. doi: 10.1186/s12263-018-0600-z (PMC5892037; doi:10.1186/s12263-018-0600-z)
Supplement: Supplementary file 3 — Differentially expressed genes associated with plasma n-3 level and expressed by more than one probe. (DOCX 16 kb) [file 12263_2018_600_MOESM3_ESM.docx]

Table S3. Differentially expressed genes associated with plasma n-3 level and expressed by more than one probe

| Gene | Highest tertile (n 18) | | Lowest tertile (n 18) | | Mean difference | P |
| --- | --- | --- | --- | --- | --- | --- |
| SNX5_1 | 7.29 | 0.20 | 7.17 | 0.14 | 0.12 | 0.054 |
| SNX5_2 | **8.53** | **0.13** | **8.43** | **0.14** | **0.10** | **0.029** |
| RORA_1 | 7.28 | 0.15 | 7.34 | 0.12 | -0.06 | 0.195 |
| RORA_2 | **7.11** | **0.13** | **7.21** | **0.10** | **-0.10** | **0.018** |
| FAM13A_1 | 6.98 | 0.08 | 6.98 | 0.08 | 0.00 | 0.986 |
| FAM13A_2 | **7.33** | **0.14** | **7.43** | **0.15** | **-0.10** | **0.049** |
| FAM13A_3 | 7.40 | 0.15 | 7.44 | 0.18 | -0.04 | 0.523 |
| UBE2L3_1 | 8.14 | 0.20 | 8.11 | 0.18 | 0.03 | 0.687 |
| UBE2L3_2 | **7.16** | **0.16** | **7.28** | **0.17** | **-0.12** | **0.035** |

Expression of genes is given as mRNA level. Values are presented as mean ± SD and are log2 transformed. Differences between tertiles were analysed using the Independent Samples t-test. P-values <0.05 were considered significant. The probes in bold are shown in Table 4.
