# Supplementary material for: Plasma fatty acid levels and gene expression related to lipid metabolism in peripheral blood mononuclear cells: a cross-sectional study in healthy subjects
Source: Genes Nutr. 2018 Apr 10;13:9. doi: 10.1186/s12263-018-0600-z (PMC5892037; doi:10.1186/s12263-018-0600-z)
Supplement: Supplementary file 4 — Differentially expressed genes associated with plasma SFA to PUFA ratio and expressed by more than one probe. (DOCX 17 kb) [file 12263_2018_600_MOESM4_ESM.docx]

Table S4. Differentially expressed genes associated with plasma SFA to PUFA ratio and expressed by more than one probe

| Gene | Highest tertile (n 18) | | Lowest tertile (n 18) | | Mean difference | P |
| --- | --- | --- | --- | --- | --- | --- |
| FAM117B_1 | 10.34 | 0.22 | 10.23 | 0.25 | 0.11 | 0.176 |
| FAM117B_2 | **8.58** | **0.27** | **8.36** | **0.23** | **0.22** | **0.012** |
| UBE2L3_1 | 8.12 | 0.22 | 8.17 | 0.19 | -0.06 | 0.398 |
| UBE2L3_2 | **7.24** | **0.18** | **7.09** | **0.13** | **0.15** | **0.010** |
| ERLIN2_1 | **6.99** | **0.07** | **6.93** | **0.07** | **0.05** | **0.034** |
| ERLIN2_2 | 7.02 | 0.08 | 6.99 | 0.09 | 0.03 | 0.269 |
| ERLIN2_3 | 7.67 | 0.12 | 7.70 | 0.10 | -0.03 | 0.400 |
| TOM1_1 | 9.60 | 0.20 | 9.73 | 0.28 | -0.13 | 0.118 |
| TOM1_2 | **7.04** | **0.08** | **7.13** | **0.10** | **-0.09** | **0.006** |
| ERGIC3_1 | 8.69 | 0.18 | 8.69 | 0.19 | 0.00 | 1.000 |
| ERGIC3_2 | **10.54** | **0.19** | **10.67** | **0.13** | **-0.12** | **0.031** |
| KLHL8_1 | 8.20 | 0.15 | 8.14 | 0.27 | 0.06 | 0.411 |
| KLHL8_2 | **8.96** | **0.21** | **9.13** | **0.25** | **-0.17** | **0.042** |

Expression of genes is given as mRNA level. Values are presented as mean ± SD and are log2 transformed. Differences between tertiles were analysed using the Independent Samples t-test. P-values <0.05 were considered significant. The probes in bold are shown in Table 5.
